# Atmospheric Sampling on Ascension Island Using Multirotor UAVs

**DOI:** 10.3390/s17061189

**Published:** 2017-05-23

**Authors:** Colin Greatwood, Thomas S. Richardson, Jim Freer, Rick M. Thomas, A. Rob MacKenzie, Rebecca Brownlow, David Lowry, Rebecca E. Fisher, Euan G. Nisbet

**Affiliations:** 1 Department of Aerospace Engineering, University of Bristol, Bristol BS8 1TR, UK; colin.greatwood@bristol.ac.uk; 2 School of Geographical Sciences, University of Bristol, Bristol BS8 1SS, UK; jim.freer@bristol.ac.uk; 3 Cabot Institute, University of Bristol, Bristol BS8 1SS, UK; 4 School of Geography, Earth & Environmental Sciences, University of Birmingham, Birmingham B15 2TT, UK; r.thomas@bham.ac.uk (R.M.T.); A.R.Mackenzie@bham.ac.uk (A.R.M.); 5 Royal Holloway, University of London, Egham TW20 0EX, UK; Rebecca.Brownlow.2009@live.rhul.ac.uk (R.B); d.lowry@es.rhul.ac.uk (D.L.); r.fisher@es.rhul.ac.uk (R.E.F.); e.nisbet@es.rhul.ac.uk (E.G.N.)

**Keywords:** Ascension Island, atmospheric sampling, methane, UAV, SUAS, multirotor, BVLOS

## Abstract

As part of an NERC-funded project investigating the southern methane anomaly, a team drawn from the Universities of Bristol, Birmingham and Royal Holloway flew small unmanned multirotors from Ascension Island for the purposes of atmospheric sampling. The objective of these flights was to collect air samples from below, within and above a persistent atmospheric feature, the Trade Wind Inversion, in order to characterise methane concentrations and their isotopic composition. These parameters allow the methane in the different air masses to be tied to different source locations, which can be further analysed using back trajectory atmospheric computer modelling. This paper describes the campaigns as a whole including the design of the bespoke eight rotor aircraft and the operational requirements that were needed in order to collect targeted multiple air samples up to 2.5 km above the ground level in under 20 min of flight time. Key features of the system described include real-time feedback of temperature and humidity, as well as system health data. This enabled detailed targeting of the air sampling design to be realised and planned during the flight mission on the downward leg, a capability that is invaluable in the presence of uncertainty in the pre-flight meteorological data. Environmental considerations are also outlined together with the flight plans that were created in order to rapidly fly vertical transects of the atmosphere whilst encountering changing wind conditions. Two sampling campaigns were carried out in September 2014 and July 2015 with over one hundred high altitude sampling missions. Lessons learned are given throughout, including those associated with operating in the testing environment encountered on Ascension Island.

## 1. Introduction

This study reports the successful development of an important new sampling technique for atmospheric methane in the mid-troposphere. Methane is a major greenhouse gas, which is rising rapidly, particularly in the Tropics [1,2]. The reasons for the rise remain unclear, but tropical wetlands may be a major contributor [3]. These wetlands, many of which are in the Congo and Amazon basins, are relatively inaccessible to integrating studies of emissions: field access is difficult, and in some regions of Africa, aircraft surveys are both challenging and not favourably viewed by local security forces. Furthermore, such integration of the more global atmospheric signal is problematic where sampling might occur near a range of source areas and convection processes.

Therefore, to obtain a more representative global-scale signal, sampling is preferred in zones that are downwind of mixed regional emissions that can then be benchmarked against local background conditions not attributed to the sources being characterised. One such unique place for such a sampling laboratory is Ascension Island in the South Atlantic. In order to sample the atmosphere downwind, a very promising research sampling platform based on a Small Unmanned Air System (SUAS) has a number of benefits. The challenge is to realise safe, repeatable and reliable operations to a significant altitude that is Beyond Visual Line Of Sight (BVLOS). This paper describes the successful demonstration of the use of SUAS to sample equatorial air up to almost 3000 m Above Sea Level (ASL) on Ascension Island, detailing the SUAS used, key operational requirements and lessons learned throughout the build-up and during the field campaigns. Using SUAS, greenhouse gas measurement on Ascension can in principle access both air at ground level from a very wide swathe of the southern oceans and also sample air from above the Trade Wind Inversion, thereby addressing emissions from a significant part of the global tropical land masses.

SUAS and UAVs are increasingly being developed and deployed for a range of environmental applications [4,5,6,7,8,9]. In particular, significant traction is being realised in the areas of remote sensing [10,11], mapping 2D/3D structures [12,13,14] and atmospheric sampling [15,16,17,18,19,20,21,22,23,24,25] using a range of emerging sensor technologies [26,27,28,29]. However, most applications to date that have been used for atmospheric sampling have been at lower altitudes in the 500 m–1000 m range [15,19] or involve longer range fixed wing platforms that often require considerable resources to deploy [30,31,32]. This paper critically evaluates the development and deployment of a multirotor-based system to investigate the feasibility of collecting mid-tropospheric air samples from above the Trade Wind Inversion (TWI) layer on Ascension Island for the purposes of identifying methane mole fractions and isotopic composition. The objectives were to capture air samples from below, within and above the inversion layer above Ascension Island in the mid Atlantic. The minimum altitude requirement was to be sufficiently above the TWI layer - which changes altitude seasonally and has some daily fluctuations - to ensure that the air captured was free of boundary layer air from below the TWI that may have mixed upward. Therefore, the higher the sampling altitude achieved, the more confidence that we can determine the mixed air back-trajectory [33,34] “reach” of the method, potentially sampling wide source regions in Africa in the right synoptic conditions.

## 2. Campaign Field Site

**Figure 1:**
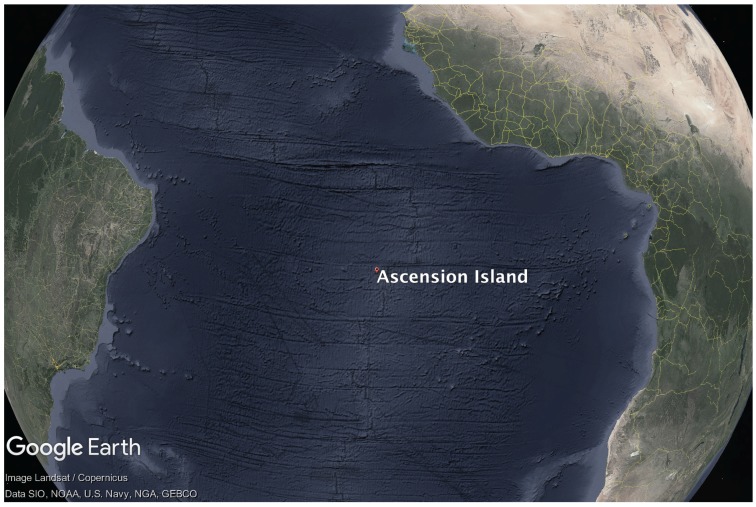
Ascension Island location in the mid-Atlantic.

**Figure 2:**
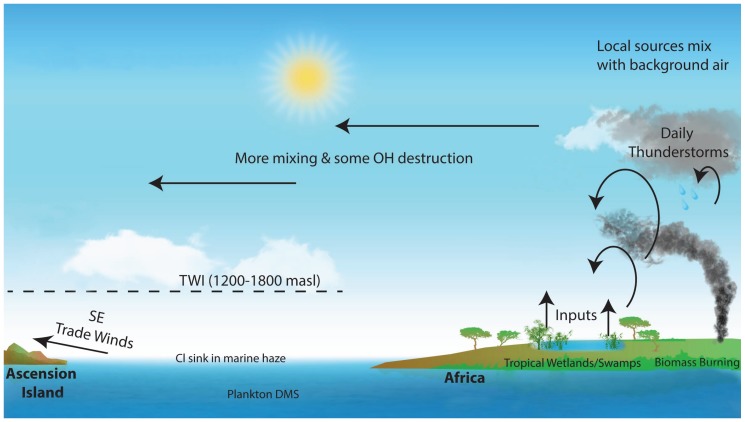
Ascension atmospheric sources.

Ascension Island, situated in the mid-Atlantic just south of the Equator (8° South) (Figure 1), approximately 1500 km from Africa, is ideally located. At sea level, the SE Trade Winds are in the South Atlantic marine boundary layer. The Trade Winds themselves are almost invariant, derived from the deep South Atlantic and with little contact with Africa. Above the TWI at about 1200 m–2000 m above sea level (depending on seasonal meteorology and diurnal cycle), the air masses are very different, of equatorial origin; see Figure 2. Dominantly, they have been last in contact with the ground in tropical Africa, but at times from South America. In detail, depending on season, air above the TWI is sourced mainly from tropical and southern Africa with some inputs of air also from southern tropical South America. African and South American methane sources are major contributors to the global methane budget [3,35], but although local campaign studies have been made, these emissions are not well known in bulk. Understanding the changing greenhouse gas burden of the atmosphere demands sustained long-term measurement. Ascension is ideal for this, both in location and in security of access.

The island hosts one of the very few equatorial high precision measurement facilities for CO${}_{2}$ and CH${}_{4}$, worldwide; see: [3]. The cavity ring-down greenhouse gas analyser and calibration suite are installed at the UK Met Office base at the Airhead on Ascension and, in normal operation, continuously measure CO${}_{2}$ and CH${}_{4}$ in the ambient marine boundary air of the Trade Wind. However, the highest point on Ascension is the top of Green Mountain, which is 859 m above sea level and is therefore not high enough to sample air above the TWI. Thus, the purpose of this SUAS project, by demonstrating the usefulness of the instrument in measuring air samples from above the TWI, was to show that Ascension potentially becomes a virtual mountain with access to air from sea level to nearly 3000 m: it can become the UK equivalent of the USA Hawaiian observatory on Mauna Loa, at 3397 m above the Pacific, if an effective SUAS sampling platform is fully demonstrated. In addition to being an ideal location for the sample flights from a science perspective; the remote location, military air base and size of the island mean it is possible to arrange for segregated airspace. This is a key requirement for allowing current SUAS to operate Beyond Line of Sight (BLOS).

## 3. Rationale for the SUAS Platform

Key targets of the field campaign and the proof of concept system can be identified as:

1. To be able to operate on Ascension Island, with the required associated logistics and support.
2. To be able to operate in a tropical equatorial environment, in close proximity to the sea, at high altitude (for SUAS) and with wind speeds averaging 8 ms${}^{-1}$ at ground level.
3. To be able to sample repeatedly at a minimum altitude 100 m or more above the inversion layer, identified on Ascension as varying seasonally between 1200 m and 2000 m ASL through the year.
4. To be able to identify the lower and upper boundaries of the inversion during flight in order to be able to target samples within, above and below.
5. To be able to remotely trigger the air sample collection.
6. To be able to fly multiple times per day, nominally six samples per day at specified altitudes in a safe and reliable manner.

The SUAS approach presented here was chosen because of its inexpensive flexibility. In previous work by the Royal Holloway, University of London (RHUL) group, air sampling has been carried out at altitude by using the UK Facility for Airborne Atmospheric Measurements (FAAM) aircraft facility to fly air sampling equipment at the required altitudes. Sampling with full size aircraft enables greater flexibility than land-based measurements offer due to the ability to climb to required altitudes, but incurs very high costs, especially in remote locations. Moreover, the FAAM aircraft barely has the range to reach Ascension with a full load of instruments. The frequency at which samples may be collected would also be very low. Whilst Ascension Island hosts the 3000 m runway of Wideawake Airfield, most flights are large aircraft en route to the Falklands, and it is not suitable for sustained sampling. No commercial light aircraft or helicopters are based on the island that could be used for the air sampling campaign. In contrast, SUAS have the potential to offer fast turn-around times, remote deployment with small teams and inexpensive sampling [36].

A number of different aerial solutions to the atmospheric sampling problem were considered, including kites, helikites and fixed-wing SUAS; however, a combination of flexibility, low cost and potential ease of operation led to the choice of a small electric unmanned multirotors. Although other options could have been made to work, the electric multirotor could fly directly to the altitude required, sample and return to base, pausing only for the sample collection at altitude. Key advantages of the multirotor were identified as:

- Potentially low cost.
- Flights could be carried out in a matter of minutes, thereby accounting for rapid changes in conditions and allowing for multiple samples at specific times throughout the day.
- Flight profiles can be near vertical, allowing for easy airspace integration and de-confliction.
- Transport and ground support for the vehicles are relatively easy to deploy.
- Maintenance is relatively easy due to a modular design.
- The design allows for flexibility in the payload integration.

There are, however, key challenges to operating a small electric SUAS in this way. These include the requirement to fly what is defined as BVLOS (Beyond Visual Line of Sight); the requirement to fly through saturated air to allow sampling above the lower cloud layers; the requirement to climb and descend at relatively rapid rates of 5 ms${}^{-1}$ continuously; the requirement to continuously monitor temperature and humidity to allow clear identification of the temperature inversion and the requirement to operate in relatively high wind speeds. Throughout this paper however, it is shown that these challenges can be overcome, and in the right situations and conditions, a SUAS multirotor is an excellent vehicle for sensing applications to over 3000 m. Two sampling campaigns were carried out in September 2014 and July 2015 with over one hundred high altitude sampling missions. Lessons learned are given throughout, including those associated with operating in the harsh environment encountered on Ascension Island.

## 4. System Description

**Figure 3:**
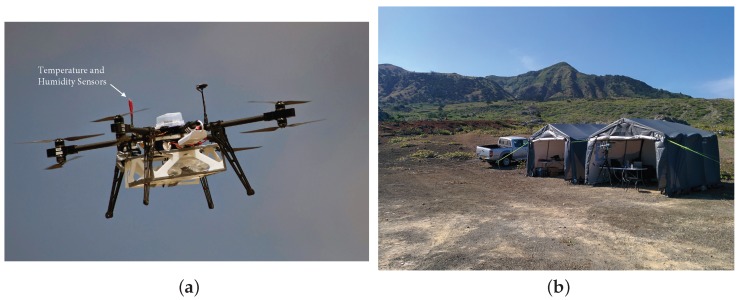
Flight operations on Ascension Island (**a**) University of Bristol X-8 Multirotor; (**b**) Field site.

The aircraft used for the sampling campaign was an eight motor multirotor (or octocopter) in an X-8 configuration, as shown in Figure 3a. The airframe is a custom design from the University of Bristol that provides enough space for a large battery capacity (typically 533 Wh, but tested with up to 710 Wh) and air sampling equipment. Situated above the main aircraft, the temperature and humidity sensors are located towards the centre and away from the body in order to minimise the effect of the local flow on the sensor readings. The Tedlar bags are held underneath the vehicle to allow for inflation at the selected altitudes. A summary of the vehicle specifications is given in Table 1, and additional key features are described as in the following section. The ground-based element of the system is shown in Figure 3b and is indicative of the operating environment encountered. In close proximity to the sea and with near constant wind speed, this required significant weatherising of the onboard electronics.

### 4.1. Airborne Vehicle

Vehicle configuration: An eight-rotor vehicle was chosen to achieve reasonable redundancy against loss of a motor or speed controller during flight. The vehicle could theoretically sustain a loss of four motors, provided that none were on the same arm, and tests on disconnecting three motors demonstrated that the vehicle retained good control in flight provided there was sufficient overall thrust. Users of octocopter platforms have also anecdotally suggested that the four-arm coaxial configuration was likely to provide better gust tolerance than a flat eight-arm configuration. It was found that the vehicle did perform well in the wind, but further research is being conducted at the University of Bristol in order to quantify the differences.

Vehicle size: The size of the vehicle was driven by three factors: the mass of the payload; the endurance required to reach high altitudes; and overall vehicle stability in high winds. Tests showed that the final flight vehicle could operate in wind speeds of up to 20 ms${}^{-1}$. The payload required to conduct the experiments was designed to be under 0.5 kg, and the maximum take off weight given in the risk assessment and the application for BVLOS operations was 10 kg. This was never exceeded on Ascension, and the typical take-off weight was in the region of 8.5–9.5 kg depending on the number of batteries that were used.

Battery capacity: The vehicle was configured to be able to operate with either two, three or four 8 Ah 22.2 V Lithium Polymer (LiPo) batteries in parallel. Fewer installed battery packs would lead to a lighter more agile vehicle, but with reduced endurance. Installing more batteries increases the flight time, but due to the additional mass, the additional endurance reduces with each battery. Four batteries was deemed as an acceptable upper limit in terms of endurance achieved and stability of the aircraft. During the campaign, the vehicle flew with three batteries, as this provided enough endurance to reach beyond the maximum expected TWI altitude of 2.0 km ASL.

Autopilot: The ArduCopter autopilot software was selected due to its reliable performance and flexible operation. The telemetry protocol is well documented and enabled integration with a long-range telemetry link. Additionally, a comprehensive flight dataset is logged for each flight, enabling the analysis presented with this paper. The hardware selected was the Pixhawk by 3D Robotics.

**Figure 4:**
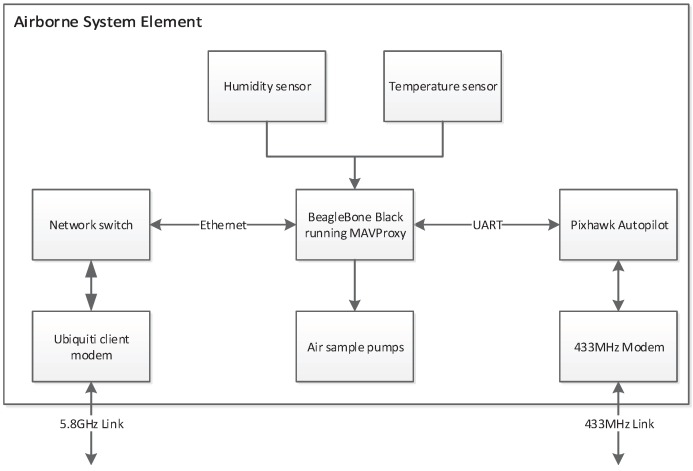
Airborne system diagram.

Onboard computing: An onboard computer was required to store and forward the analogue and digital data from the onboard sensing, as well as control the sample collection pumps. This enables the ground operators to see the sensor data in real time for decision making in flight. MAVProxy software was installed for forwarding the telemetry data, and a custom module was written to allow integrated monitoring and control of the payload. All collected data were stored on-board to allow for dropped packets. The architecture of the on-board system components and how they communicate is shown in Figure 4.

Sensors: The payload had three core functions: measure temperature; measure relative humidity; and pump air into sample bags from an external demand signal. Temperature and humidity measurements were used to characterise the boundary layer profile on the ascent and to indicate the location and characteristics of the inversion layer. The ascent rates were reasonably fast (5 ms${}^{-1}$), and so it was necessary to select sensors that responded fast enough in order to measure the boundary layer height. To target a minimum of 100 m above the trade wind inversion when ascending at this rate, at least one of the sensors needs to be fast enough to respond to a step change in temperature within 20 s for this to be the case. Ideally, this is within 10 s to account for uncertainty in the down leg measurement when assessing the sample height post-flight (we do not profile up and down and then choose a sampling height), and we test this for the temperature sensor using derived boundary layer heights in Section 8.

**Figure 5:**
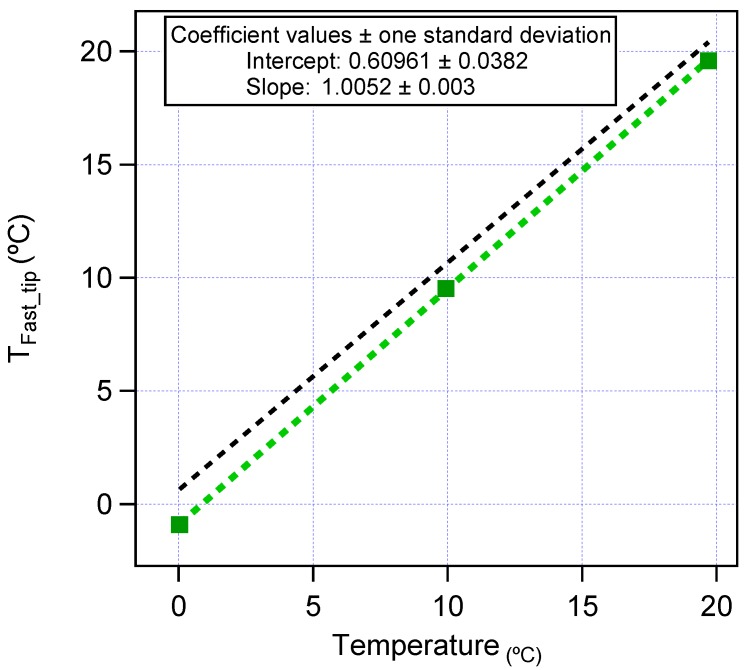
Temperature sensor calibration.

Sensors used in radiosonde measurements were sourced due to having very similar design requirements. The temperature sensor was easy to interface through the onboard computer analogue inputs and proved to be reliable; however, the relative humidity sensor required more attention to integrate due to the limited I2C address range available, and custom addresses had to be programmed ahead of time. The temperature sensor itself was a GE fast-tip FP07 glass bead thermistor (analogue) <0.2-s response time with a spectral response close to 5 Hz (Figure 15 in [37]). Figure 5 shows a three-point calibration for this sensor in a standard Weiss WKL 34/40 calibration oven. The calibration was performed at three set points (0 °C, 10 °C, 20 °C), and the reference was a NIST traceable temperature logger combined with a standard thermistor probe (accurate to ±0.2 °C). Based on this data and in the configuration used, this was found to be accurate to ±1 °C and therefore suitable for the purposes of this campaign.

The relative humidity sensor was significantly more sensitive to general handling and dust ingress than the temperature sensor. The reliability of the sensor was poor, and so, it was replaced daily and protected between flights. The humidity sensor used was an IST P-14 Rapid capacitance humidity sensor (I2C) <1.5 s response time, which has been extensively tested for fast-response humidity measurements on UAVs by Wildmann et al. [38]. As with the Fasttip temperature sensor, we expect the humidity sensor characteristics to satisfy our minimum 100 m vertical resolution requirements, as mentioned above. The manufacturer states that these are calibration free following their factory calibration to ±3% relative humidity, and no changes were made to the sensors themselves prior to flight.

As mentioned previously, the temperature and humidity sensors were placed toward the centre of the vehicle and above the main chassis. This was done to minimise the effect of the rotor wake on the sensor readings, specifically in the climb to the sample altitudes. Whilst ascending, the air is essentially accelerated vertically downwards, and the sensors themselves are in the relatively smooth air being induced from above (Figure 3 in [39]). It was also found that inspection of the data collected during ascent and descent showed no systematic bias with regards to temperature and pressure. We conclude, therefore, that rotor-induced local air flows have no significant effect on the performance of these sensors when placed in this position. In addition, with the response time of the temperature sensor used at <0.2 s, the resolution of the data collected at (5 ms${}^{-1}$) ascent rate was higher than required in order to pinpoint the temperature inversion and the sample heights to target during the flight. It was also found that when a second flight was carried out in quick succession to a first flight, the temperature and humidity data followed the first very closely.

Air collection: The air samples were collected into five-litre Tedlar bags by directly pumping in air through NMP 850 KNDC diaphragm pumps, in accordance with internationally-agreed best practice [3]. Two pumps were installed, each plumbed directly to a sampling bag. The pumps were controlled via a P-Channel MOSFET load switch circuit triggered from the onboard computer. Whilst the pumps could have been triggered automatically upon the vehicle reaching specific waypoints, it was decided that the triggering should happen remotely, allowing the payload operator the chance to decide during flight on the best sampling locations. A custom box was laser cut out of corrugated correx to house the two bags and was simply attached to the underside of the vehicle. Methane mixing ratios in the Tedlar bag samples were measured within 1–2 days of collection using an in-house Picarro 1301 CRDS (cavity ring-down spectrometer) with an NOAA traceable six-gas calibration suite (on Ascension Island) giving a precision of ±0.5 ppb [40]. Samples are measured for 240 s with the last 120 s being used to determine the mixing ratio. For bags containing less air, the bags were run for 120 s, with 60 s being used to determine the mixing ratio.

With regards to mixing of the air sample due to the vehicle rotors, the induced velocities at a distance of greater than three rotor diameters are very low (note that this does vary depending on disk loading [39]), and so, although the air is mixed locally due to the air vehicle whilst in the hover and when collecting an air sample, the volume over which that sample is collected is actually relatively small, with an outer sample collection diameter (in the absence of wind) of no more than a few metres. With the vertical distances of up to 3 km involved in this campaign, the sample volume itself is relatively very small. With the vehicle itself drawing in air for the sample from only a few metres, the atmospheric conditions themselves will typically have a much greater effect on the mixing of the sample than the vehicle itself.

The air masses under study have been transported several thousand km from interior Africa and have mixed en route. Thus, though on 3000 m scale, there are strong vertical changes depending on source inputs; each air mass is generally homogeneous on the 10–50 m scale. The UAV causes local mixing on a metre scale, but this scale of mixing is unlikely to be significant in sampling separate air masses, unless there is a sharp laminar boundary present that has survived the transport from Africa and consequent boundary mixing between air masses.

Tedlar bag samples were also taken approximately 1 m above ground level each day from the UAS site and were analysed together with the samples from the UAS. These samples were then compared with the continuous ground measurements made by RHUL at the Met Office on Ascension Island. The measurements by RHUL are long-standing, both with the in situ continuous system installed on the island and by regular flask sampling analysed in London. RHUL measurements are subject to ongoing intercomparison with the parallel co-located flask collection by U.S. NOAA, measured in Boulder Colorado. Please see [3] for additional information.

Safety pilot link: The safety pilot link is used for manual control of the aircraft, as well as selecting flight modes, such as waypoint following or Return To Home (RTH). The link is required to maintain communication with the vehicle at all times so that the safety pilot could always command an RTH. Two off-the-shelf systems were identified as suitable for the safety pilot link: Immersion RC EzUHF and FrSky L12R systems. The Immersion RC system transmits on 459 MHz (in the UK firmware), whilst the FrSky system uses 2.4 GHz. During testing, it was found that the Immersion RC system was sensitive to interference from the onboard systems. The FrSky system proved extremely robust, both during testing, as well as the campaign. Ground-based tests were conducted before the campaign with a line of sight horizontal separation of 3 km during which the signal strength was consistently strong.

Telemetry: Ubiquiti radio modules were selected due to the long range and high bandwidth offered. The primary function of the telemetry link was to enable monitoring of system health data on the ground, as well as interaction with the payload. The directional antennas used both on the ground, as well as the aircraft were selected to provide a stronger link. A second telemetry link was used for redundancy, transmitting on a separate frequency using a simple omnidirectional antenna. Radio modems by 3D Robotics were used to communicate with the vehicle on 433 MHz, with duplicate vehicle health information being transmitted.

Environmental protection: The motors are able to operate in water and did not require special consideration other than inspection of bearing smoothness prior to flight. Electronics however, required protection, and the onboard computer was sprayed with PCB lacquer; both the autopilot and onboard computer were encased within plastic containers with vents on the underside. The vents would enable air to come in, which would contain moisture from the clouds, but signs of any water deposited within the containers was closely monitored and found to be minimal. Connecting wires were sealed, and loops to below the entry points were included to minimise the run down of collected water droplets.

### 4.2. Ground Station

**Figure 6:**
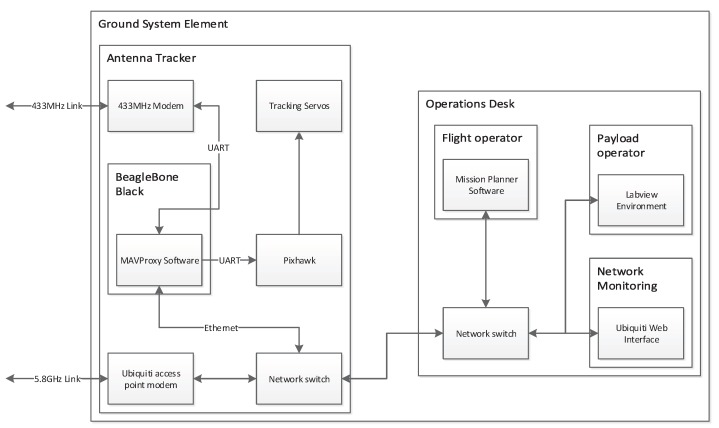
Ground system diagram.

A ground station network was set up as shown in Figure 6. This was designed in order to provide the mission and payload operators with the information required to operate the vehicles safely and reliably. Key elements of this system are as follows.

Antenna tracker: A servo-driven pan and tilt system was constructed with both the 433 MHz and 5.8 GHz antennas attached. A Pixhawk running the ArduTracker software was installed to control the antenna tracking, which compares the UAV position with the position and attitude of the antennas and sends corrective signals to the pan and tilt servos. The Pixhawk obtains information about the position of the UAV from the MAVProxy software, which combines information about the UAV from both telemetry links. The combined information is also shared with the flight and payload operators through a two-way data link over the local area network.

Visitor and road management: Road blocks were setup each day before flight operations commenced as agreed with the Ascension Island police force. During operations, there was always at least one person ready to handle any external interruptions, allowing the rest of the team to continue focussing on the mission. Typically, this would involve answering the radio when road access was requested, but also included talking to visitors that had arranged to watch the flights.

Weather monitoring: A portable weather station was used during the first campaign, primarily to measure wind speed and direction. During the second campaign, a Gill Instruments R3 Sonic Anemometer was used, which enabled remote monitoring of the same information.

## 5. Operational Considerations

### 5.1. Field Site Operations on Ascension Island

In September 2013, a three-person team from Birmingham and Bristol visited Ascension Island for a reconnaissance trip to identify possible operational sites. This proved to be invaluable for the subsequent field campaign, and the authors highly recommend this approach for any significant UAS operations. Three possible sites were initially identified, of which one was selected as the most suitable to operate from. The area found was located by the road leading out to the old NASA site and provides excellent access, isolation and the ability to block the road and control access for third parties. It is approximately 350 m ASL, which reduces the height required to climb compared to a sea level launch and is on the windward side of the island, providing clean, unobstructed airflow from the prevailing wind direction: south, southwest.

Ideally, all of the equipment including both the vehicles and the ground support equipment would have been shipped to Ascension prior to the field campaign. Due to extended development and early shipping dates, however, only the ground equipment and maintenance equipment were sent out ahead of time. This included all of the lithium polymer batteries (necessary due to airfreight restrictions) and all the heavy items, such as portable shelters. Flights to Ascension Island depart from Royal Air Force Brize Norton, with the Air Bridge to Ascension Island and the Falkland Islands.

**Figure 7:**
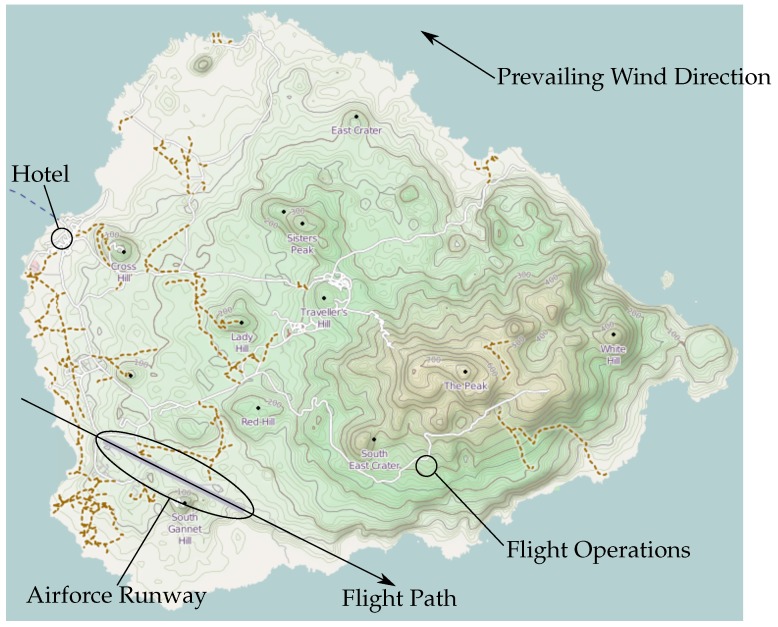
Map of Ascension Island.

With the permission of the military personnel and police on Ascension, a base was set up, as shown in Figure 7, just off the road to the old NASA site. Given the strong continuous wind speeds on Ascension, two 3.66 m (12 ft) by 3.66 m (12 ft) shelters were shipped from the UK and assembled on site. During the course of the campaign, basic equipment was stored on site throughout, with the aircraft being bought up from Georgetown on a daily basis. Battery charging was carried out both on site and in Georgetown depending on daily usage. Only minor maintenance was required throughout the campaign, and this was carried out in Georgetown.

Safety was the focus of the build-up and operations through the project. Although there is an element of redundancy through the air vehicle configuration chosen, there are still multiple single points of failure on the airframe. Because of this, all operations were carried out with the worst safety case based on a total failure of the onboard power systems. Given the maximum permitted BVLOS altitude of 3048 m (10,000 ft), the worse case scenario considered given the conditions encountered throughout the campaign resulted in a safety radius of at least 1.2 km from the flight path. The road was blocked at 1.4 km (straight line) from the point of operation, and the nearest inhabited site was 3.0 km from the flight path.

### 5.2. Typical Flight Operations

The objectives required the multirotor to climb to a commanded altitude and loiter in position whilst an air sample was taken, pausing for ten seconds prior to the sample collection. This air sample was collected using a diaphragm pump that inflated a bag in just under one minute. The vehicle would then return to base, and the air sample would be removed for analysis. Throughout the flights, the temperature and Relative Humidity (RH) were measured and transmitted to the ground station at a rate of 10 Hz, and it was possible to ascertain the location of the trade wind inversion and the different air masses from the temperature and humidity readings during the ascent. The real-time measurement of the atmospheric profile is a significant capability of the system and enabled the atmospheric scientists to accurately target key parts of the profile to collect the samples.

For both campaigns, the flights on Ascension Island were carried out under Beyond Visual Line Of Sight (BVLOS) conditions. This required an exemption to the Air Navigation Order, which was granted by Air Safety Support International (ASSI) for the period of both campaigns. The exemption itself was based on a safety case, which the University of Bristol put together, and was granted subject to a number of conditions including: contact with Wideawake Airport would be maintained at all times; all flights would be operated in accordance with the permission/procedures agreed with the Royal Air Force and the United States Air Force. These measures put in, both prior to the campaigns and during flight operations, were designed to ensure complete separation from other air traffic. For example, no flights were carried out during a given window encompassing an aircraft arriving or departing from the island. Whilst this approach to BVLOS operations requires airspace separation and close communication with all other airspace operators and users, it allows small SUAS vehicles to be operated safely in challenging environments. Ongoing improvements with communications, sensors and computing will allow more closely-integrated BVLOS operations in the future; however, for the present, the use of segregated airspace for these types of operational flights is likely to be required.

**Figure 8:**
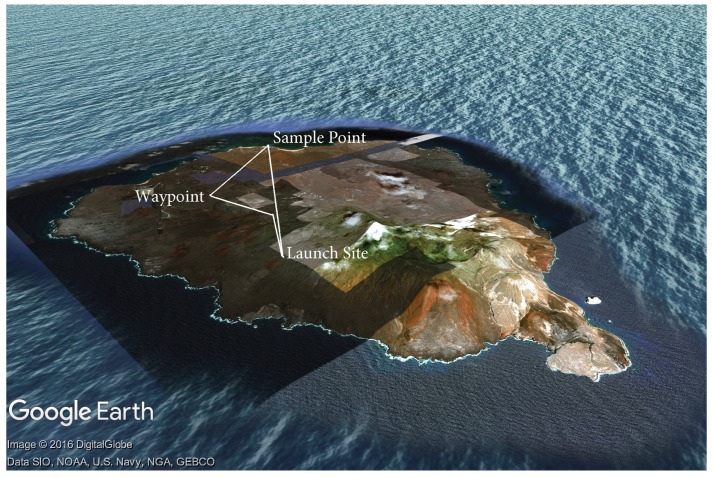
Sample flight path over Ascension Island.

The flight operations themselves followed a pattern, established over the course of the first week. This was based on the following tasks:

1. Preparation of the flight vehicle including physical and system checks and preparation of the air sample bags including air evacuation.
2. Preparation of the flight plan, including alternative routes for descent depending on the upper wind conditions. See Figure 8 for a visual depiction of a typical mission.
3. Pre-flight checks, arming the vehicle and manual take-off to 20 m AGL undergoing manual flight checks by the safety pilot.
4. Switching the vehicle into automatic mode and carrying out the ascent to the pre-determined altitude at 5 ms${}^{-1}$ ascent speed.
5. Monitor temperature and relative humidity profiles throughout the climb, confirming the location and thickness of the trade wind inversion. The sensor operator at this time would confirm the predetermined sample heights or adjust depending on the altitude at which the trade wind inversion was encountered.
6. On agreement with the ground station and sensor operators, the air samples were triggered at the required altitudes. Pump times varied between 40 s and 60 s depending on the target altitudes for the samples.
7. The descent was carried out automatically at −5 ms${}^{-1}$, reverting to manual at 20 m Above Ground Level (AGL).
8. Post flight checks were then carried out, battery voltages recorded, flight data stored and the air sample(s) retrieved.

For all flights on Ascension Island, the aircraft was taken off and landed manually, under the direct control of the safety pilot. Although the system is fully capable of an automatic take-off and landing, a manual approach allowed for the safety pilot to carry out flight checks prior to initializing the mission. The take-off point itself was situated across and downwind from the operations tent, allowing the ground crew to remain upwind and yet control full access to the site. One of the benefits of operating on Ascension Island is that the prevailing wind direction and strength at ground level are consistent and predictable, allowing the flight operations to be consistent and refined over time.

## 6. Lessons Learned

All anomalies with any part of the system were fully investigated prior to any operations. The following is a list of the key problems encountered during the field campaign and the actions taken as a result.

1. Ground station power was lost during one of the initial flights. The power sources taken to support operations malfunctioned, and a return to launch was triggered by the safety pilot. All subsequent operations in the first campaign were powered from two inverters. For the second field campaign, a generator was sourced to provide power on site.
2. One of the ESCs (Electronic Speed Controller) failed as the aircraft was prepared for flight. This was identified by the safety pilot and replaced before further operations continued.
3. Difficulties were identified at one point with the telemetry downlink; this was traced to a file size limit being reached on the onboard computer. Once identified, this was corrected, and flights continued.
4. High wind speeds above the inversion were identified in the second half of the first field camp again. These are outlined in detail together with mitigation strategies in the subsequent section.
5. One of the GPS batteries became loose during flight and was identified in the pre-flight checks prior to the following operation. This was triggered as a magnetometer error, and the unit was replaced and checked before the subsequent flight.
6. One of the commercially-purchased battery connections failed during flight, which was identified from the flight director observing an unusually high voltage drop on the initial climb out; therefore, a return to home was triggered by the safety pilot. All additional battery connections were checked and modified prior to resumption of flight operations.

## 7. Flight Envelope

### 7.1. Achieving Maximum Altitude and on-Board Power Management

**Figure 9:**
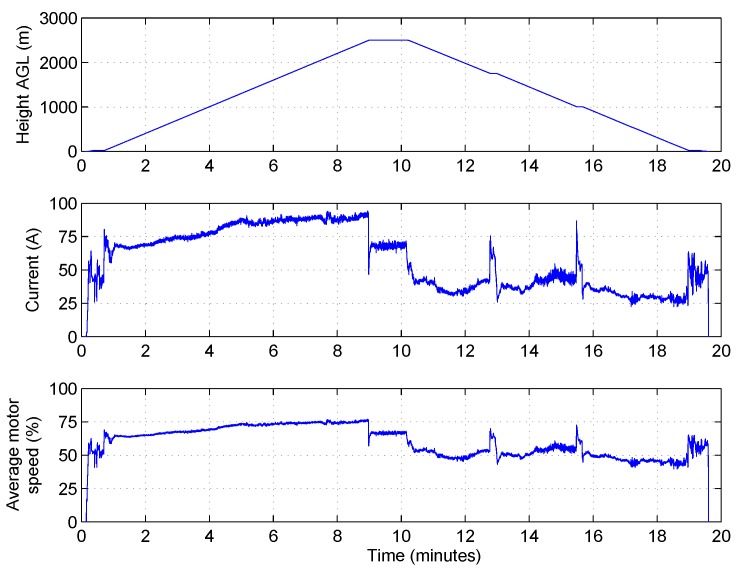
Altitude, battery voltage and motor speeds for a sample flight to 2500 m AGL.

At the start of the campaign, the maximum commanded altitude of the UAV was increased gradually as knowledge and confidence in the system grew. The maximum altitude achieved was 2500 m AGL, which surpassed the required altitude (typical of the local TWI) by approximately 1000 m. Figure 9 shows some of the recorded data for a 2500 m AGL flight. The current drawn from the flight battery illustrates that, as one might expect, the majority of the energy is expended during the ascent. The UAV drew 45 A in the hover at 20 m AGL (370 m ASL) and peaked at 90 A at the top of the ascent, before dropping to just under 70 A whilst hovering at 2500 m AGL. The increase in motor speeds required to hover at 2500 m AGL is also apparent from the bottom of the plots, where the speed to hover at 20 m AGL is 50% compared to 66% at 2500 m. On this particular flight, the UAV landed with 23% of the battery capacity remaining.

### 7.2. Performance in Wind

**Figure 10:**
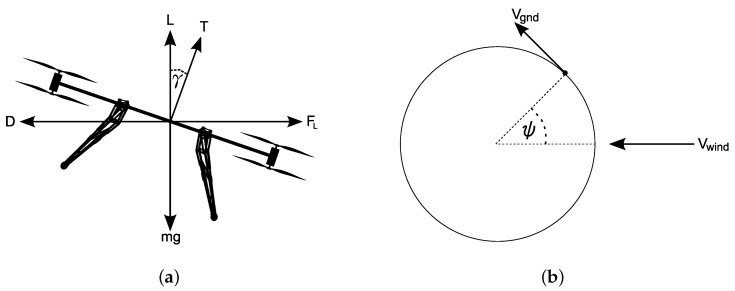
Forces, angles and velocities used for wind estimation. (**a**) Free body diagram of forces on UAV; (**b**) angle around track during orbit manoeuvre.

The trade wind conditions were found to be very consistent from day to day at ground level. The winds above the TWI, however, were seen to change over the course of the campaign. The different wind conditions therefore enabled the team to test the UAV performance in conditions ranging from light wind to prohibitively strong winds. It is possible to estimate the speed and direction of the wind from the aircraft orientation after estimating some key parameters found by performing a slow orbit manoeuvre, such as that shown in Figure 10b. Making some assumptions about steady wind conditions and resolving free body diagram forces, a simple mapping function can be created between attitude angles reported by the autopilot and the estimated wind conditions. Full details on the procedure are outlined in [41].

### 7.3. Flight through Still Air

**Figure 11:**
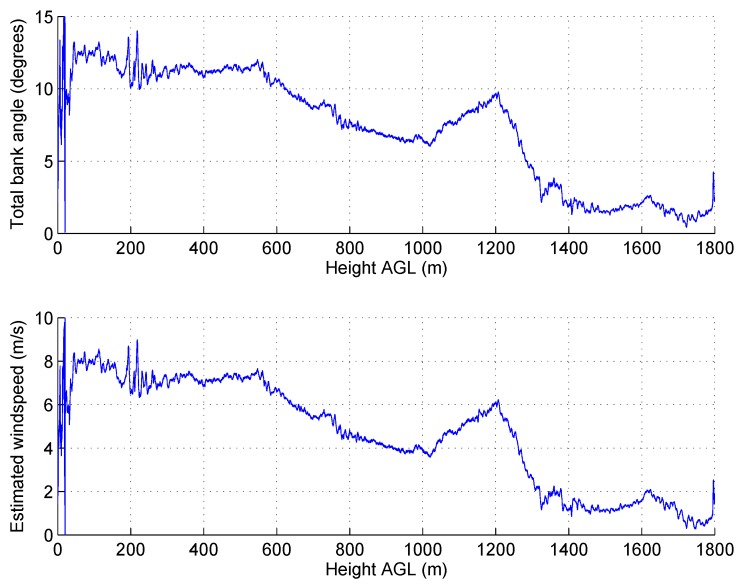
Estimated wind speeds from the aircraft attitude during a flight up to 1800 m AGL. (**a**) measured bank angle; (**b**) estimated wind speed.

The ideal flight path for the UAV from a mission scripting perspective would be to ascend vertically to the required altitude and then descend back along the same path to the take-off site. Rapid descent through still air for rotary wing aircraft however can lead to instability. The wind speeds on Ascension Island were typically around 8 ms${}^{-1}$ at ground level, which is equivalent to the UAV travelling at a reasonable forward velocity in still air conditions. As previously mentioned, the wind conditions varied above the TWI, and over the course of a few days of the first campaign, the wind speeds were found to be extremely low. Conditions varied significantly reaching less than 2.5 ms${}^{-1}$ at times, such as in the flight shown in Figure 11 where aircraft bank angles and estimated wind speeds have been plotted against height above ground.

**Figure 12:**
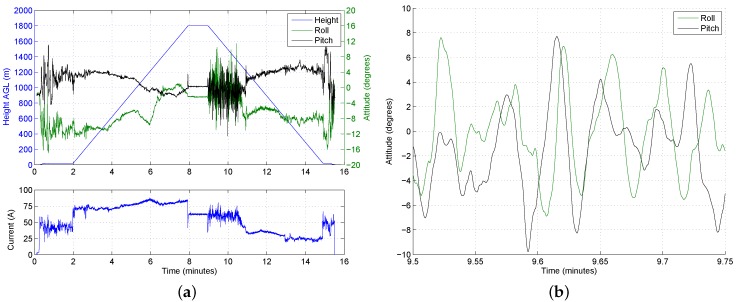
Sample at 1800 m AGL, with low wind speeds above the TWI. (**a**) Attitude and voltage profiles; (**b**) attitude during initial descent.

Figure 12a shows the roll and pitch angles during the same flight, overlaid on the altitude. The wind speed on the ground was approximately 8 ms${}^{-1}$, meaning that the vehicle had to roll and pitch in order to maintain position during the loiter and vertical climb-out. An interesting result can be seen around the four- to seven-minute mark (i.e., between 600 m and 1500 m), where the roll and pitch angles change significantly during the ascent. During this stage of the ascent, the angle of the vehicle has rotated from around $-10^{\circ }$ roll and $4^{\circ }$ pitch to approximately $0^{\circ }$ roll and $-2^{\circ }$ pitch. The change in attitude is attributed to a dramatic reduction in wind speed as the vehicle enters a different air mass.

During the ascent, the reduction in wind speed poses no problems. Descending through still air, however, requires the vehicle to constantly make corrective actions, and it has to work harder to maintain a level attitude. The attitude is shown in Figure 12b for a 15 s period half way through the still-air descent phase (at around 1600 m). Although the magnitude of the attitude variations is manageable, the persistent rate of change in attitude is undesirable. The constant sharp changes in attitude observed in the still-air descent will lower the vehicle’s endurance. Figure 12a also shows the current being drawn by the UAV, which during the initial descent through still air, is roughly the same as that required to hover at 1800 m. Upon descending into the different airmass with higher wind speeds, the current drops to nearly half as the motors do not have to work as hard to maintain the stability of the aircraft. The current only increases again at the end of the flight when the UAV hovers briefly prior to touch down.

### 7.4. Trajectory Design for Descent in Still Air Conditions

It is well known by helicopter pilots that one should not descend vertically, as this would entail entering the helicopter’s own wake and lead to instabilities as described in [42]. The descent through still air described in Section 7.3 is undesirable as it reduces the endurance of the vehicle, which in turn reduces the altitudes attainable. To provide the rotors with clean airflow, the mission script was modified to include a lateral manoeuvre during the descent. This lateral manoeuvre, referred to here as a dog-leg, typically consisted of translating away from the launch site whilst descending, pausing and then translating back towards the launch location. This can be seen clearly in Figure 8 with a climb to the sample altitude, followed by a descent to the waypoint identified, then a return to the original trajectory. The profile shown is to the same scale as Ascension Island.

**Figure 13:**
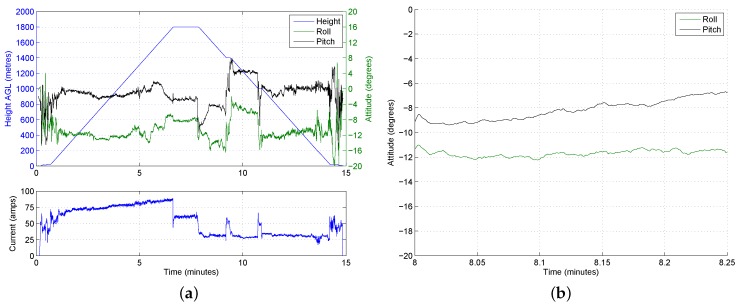
Sample flight to 1800 m AGL, with a translation manoeuvre through low wind speeds above the trade wind inversion. (**a**) Attitude and voltage profiles; (**b**) attitude during initial descent.

The current consumption shown in Figure 13a suggests that the inclusion of a dog-leg reduced the power consumed, as the current drops significantly once the descent has initiated and, apart from two brief sections of hovering, stays low. The pauses at the additional waypoints required the UAV to arrest its movement and draw additional power, but this is believed to have significantly less impact on the endurance than omitting the dog-leg and descending vertically in still air. It should be noted that the dog-leg does not increase the time taken to descend as the vertical velocity is unchanged. The key reason for including this manoeuvre however is the reduction in the roll rates that are experienced during the descent in the low speed air mass. This reduction in rolling and pitching during the initial descent can by observed by comparing those angles previously shown in Figure 12 and those found by including a dog-leg in Figure 13b.

The improvements observed by updating the flight plan with knowledge of the wind conditions suggests that some form of automatic trajectory generation would be highly beneficial. The wind direction and magnitude can be estimated using the attitude of the vehicle during the ascent, and as previously observed, the still air masses can clearly be spotted by the dramatic reduction in roll and pitch angles. An automatic trajectory planner could therefore introduce flight paths to ensure a stable descent. Furthermore, the planner should be constrained to guarantee that the trajectory does not pass into prohibited airspace and that the trajectory brings the vehicle back to the launch site.

### 7.5. Upper Wind Speed Limits

**Figure 14:**
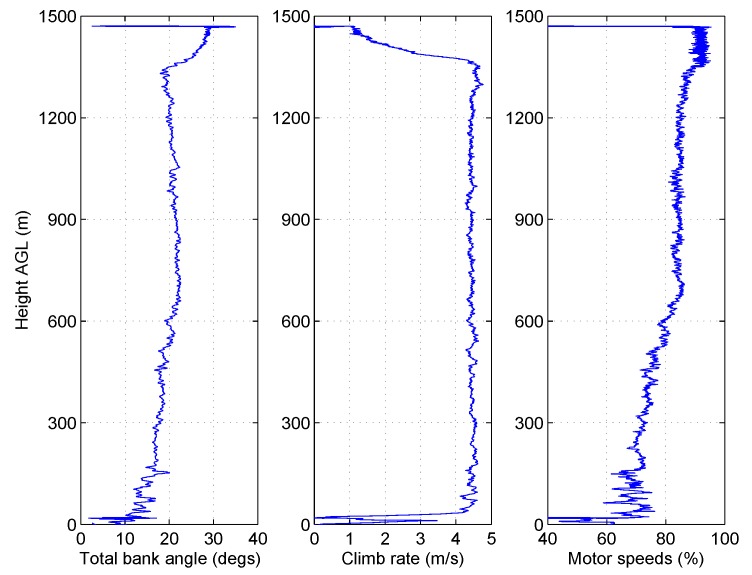
Sample flight during high wind speeds above the TWI.

The wind speeds above the TWI were, on some of the days during the campaign, observed to increase rather than decrease, as discussed in Section 7.3. Figure 14 shows flight data collected on such a day, where the wind speed increases significantly at around 1350 m AGL.

Figure 14 shows how the climb rate remained constant at 4.5 ms${}^{-1}$ until around 1350 m AGL when it was seen to decrease to just 1 ms${}^{-1}$. The decrease in climb rate was a direct result of the increase in wind speed, which caused the vehicle to pitch and roll to maintain the commanded ground course. The figure also shows the total bank angle of the vehicle during the ascent by taking the magnitude of the roll and pitch angles. The low level strong winds required the aircraft to bank by an average of 20° during the main portion of the climb. At around 1350 m AGL, however, the attitude required to maintain ground course increased to nearer 30° from level, dramatically reducing the available thrust to maintain the desired climb rate. Upon inspection of the motor speeds in the bottom of the figure, it could be reasonably assumed that the flight controller has saturated the motor outputs within the requirements for stability, i.e., the motors are spinning at their maximum speeds. Throughout these high winds, the UAV was still able to maintain an ascent, albeit at a reduced 1 ms${}^{-1}$.

## 8. Payload

### 8.1. Meteorological Sensor Assessment

The fast-response temperature and humidity sensors generally performed well during the field campaigns, and we assess their performance in terms of Targets 3 and 4. However, the normally robust capacitance RH sensors were adversely affected by volcanic wind-blown dust during the first campaign. For the second campaign, an improved shielding cap (with sufficient holes for rapid air ingress, but not allowing large dust particles to enter) was designed. The shield was constructed from white correx with an extended circular plate over a perforated tube. There is no evidence, e.g., from a discrepancy between the upward and downward legs, to suggest the shield had a significant effect on the time response of the temperature and humidity sensor system. In addition, the high wind speeds encountered during most flights aspirate the sensor to make solar heating effects unlikely [43].

**Figure 15:**
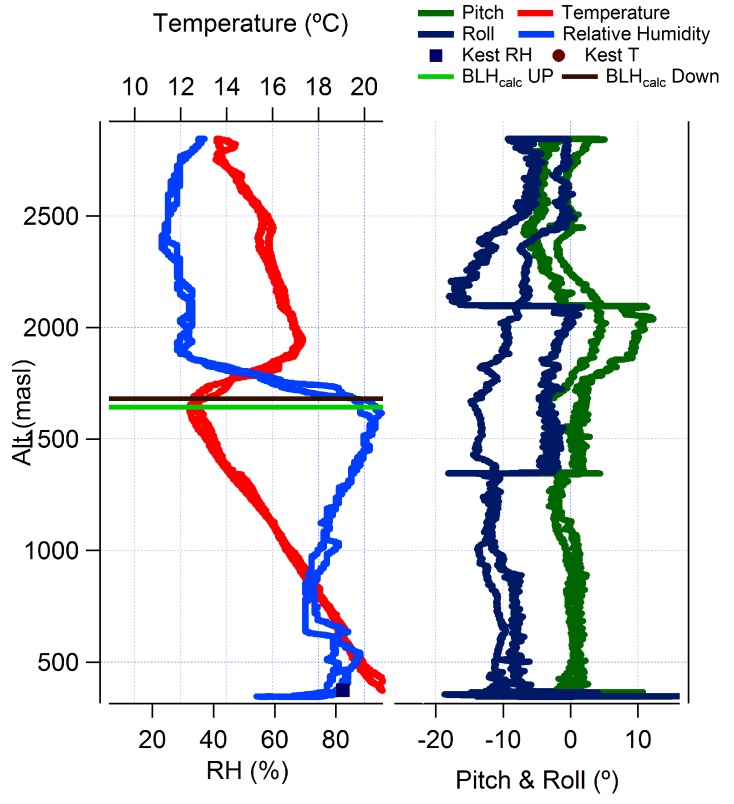
Meteorological data: temperature (red), relative humidity (light blue), pitch (green) and roll (dark blue) angles for a sample flight made at 16:54 p.m. on 14 September 2014.

Figure 15 shows a typical flight profile of temperature (red) and humidity (light blue) and also the pitch (green) and roll (dark blue) angles during a flight made on 14 September 2014. A kestrel 4500 weather station under manufacturer’s calibration was operated at the measurement site during each flight day, and these baseline measurements are shown for reference and comparison with the onboard sensors. Pitch and roll angles are included in Figure 15 to enable the reader to identify where the samples are being taken; which in this case corresponds to the two altitudes where there are rapid variations in roll and pitch angles. In this example, the base of the TWI, where temperature begins to rise with altitude, is apparent at 1670 m ASL and marks the top of the turbulently-mixed atmospheric boundary layer below. The inversion zone acts as a cap on the upward motions of the boundary layer. Figure 15 shows the temperature increasing from 12 °C–17 °C over this transition to the unmixed free-troposphere above, and correspondingly, humidity drops from near saturation in the boundary layer to 30 percent at at 1880 m ASL, the top of the TWI. This thickness of 210 m for the inversion (entrainment) zone is at the larger end of inversion thickness observations during both campaigns, with 50 m–70 m being more typical.

The temperature and humidity traces show both the upward and downward data and clearly demonstrate the suitability of the sensors for this task with a temperature difference of less than a degree and a minimal relative humidity difference for most, some of the larger differences most likely represent real variability, e.g., the proximity to clouds/proto-clouds in the lower boundary layer at about 600 m ASL. Given the narrow inversion layer thickness, this campaign clearly demonstrates the ability of small UAVs to measure small-scale atmospheric features (<200 m) with minimal disturbance. With such a system, there is potential for new insights into such features, which may have implications for numerical weather prediction and global pollutant transport models.

With the data shown in Figure 15 relayed to the ground station in real time, it was possible during the ascent to identify the exact location of the inversion in terms of the lower and upper limits. Based on these, the sample positions were then chosen, allowing the air samples to be taken in the upper air mass and clear of the inversion, within the inversion itself or below it. This capability, in the presence of uncertainty in the meteorological data prior to the flight, provided the required confidence for the selection of the air sample collection points relative to the TWI.

To demonstrate the performance of this system, the inversion height for the up and down legs of flights from September 2014 have been calculated and compared. Sensor lag time will manifest as a systematic difference in calculated boundary layer height when two profiles are compared; up-leg Boundary Layer Height (BLH) will be higher than down legs. This will give an indication of the Boundary Layer Height accuracy based on sensor lag time.

A potential temperature method was used to identify the start of the potential temperature gradient maximum, similar to Hennemuth and Lammert [44]. Methods to calculate the inversion height are varied [45] and are beyond the scope of this paper to resolve; however, using the same algorithm and thresholds to calculate the up- and down-leg boundary layer heights offers directly comparable results in this test. The result of this analysis for 25 flights that crossed the TWI in the September 2014 campaign demonstrates a positive bias; two out of 25 flights analysed demonstrating a negative difference between BLH-up minus BLH-down. The mean vertical difference was 30.7 m with a standard deviation of 21.1 m (maximum 76.5 m, minimum 2.3 m), which is well within the stated aim of targeting 100 m above the TWI.

**Figure 16:**
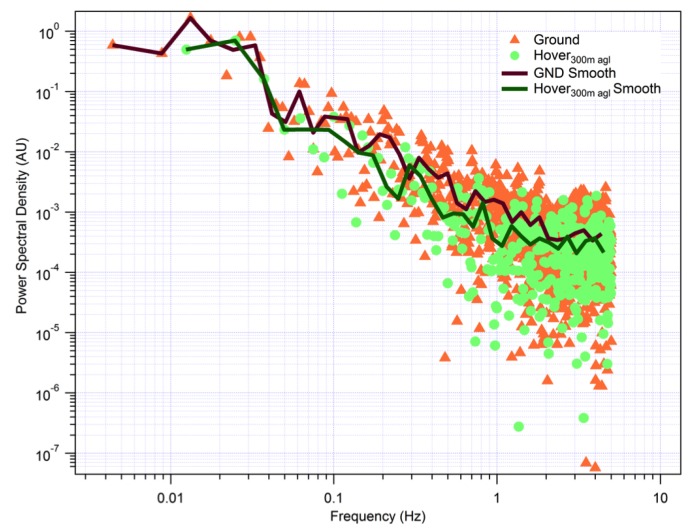
Power spectral density (arbitrary units) for the fast tip temperature sensor when stationary on the ground (motors off) and at a level hover at 300 m. Averaging periods were 226 and 80 s, respectively. Propellers rotate at 67.5–104 Hz, and this fundamental frequency is greater than the approximately 2-Hz response time seen here. Agreement between the spectra indicate no influence of either the propellers or the aircraft movement on the measured temperature.

To assess the influence of propeller interference in the sensor response, we contrast power spectra for two periods, one when the Octocopter was stationary on the surface and one when in a hover at 300 m above ground level. Figure 16 shows the raw and smoothed power spectra for each period a −5/3 line is shown for comparison. The propeller rotation rate for the motors and propellers used, Table 1, was between 4050 (50% throttle) and 6250 (100% throttle) rpm, or 67.5–104 Hz. These fundamental frequencies and associated harmonics are not seen in the temperature sensor, which from the spectra in Figure 16 responds up to 2 Hz in our system. There is also no indication that the propellers or the aircraft’s movement during hover influence the temperature signal.

### 8.2. Methane Sample Results

**Figure 17:**
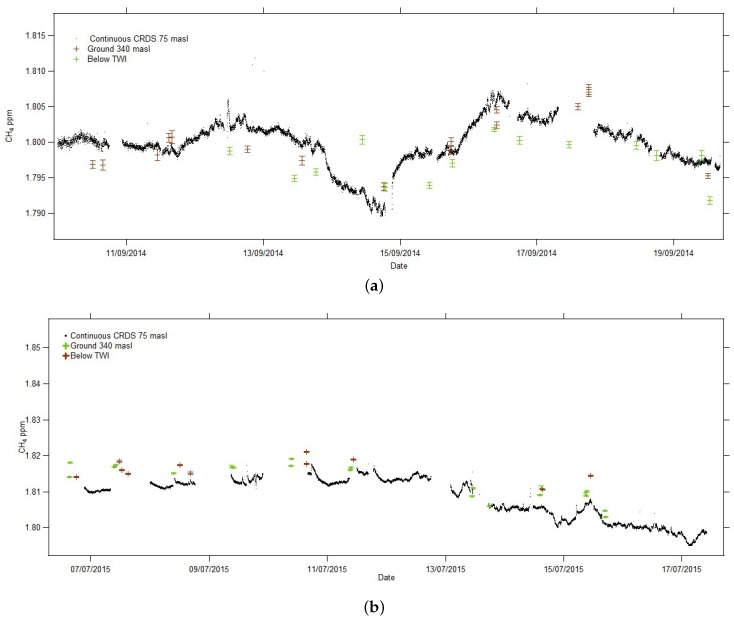
Continuous cavity ring-down spectroscopy (CRDS) CH${}_{4}$ ground level (75 m ASL) compared with bag samples taken from the ground (340 m ASL) and below the TWI. Date markers are 00:00 UTC. (**a**) 2014 campaign; (**b**) 2015 campaign.

Figure 17 demonstrates the good agreement, within expected variability and vertical profile, between the ground bag samples and the continuous values measured by the permanent cavity-ringdown system installed on the ground at the Airhead. It also shows that there is good consistency between the ground bag samples and the bag samples taken below the TWI with the r${}^{2}$ values for the two plots greater than the critical value showing significant correlation. Details for each campaign are:

Figure 17a, September 2014 campaign: correlation between the ground bag samples and CRDS samples: r${}^{2}$ = 0.558 where critical r${}^{2}$ = 0.247 at 0.05 significance. Correlation between ground samples and samples taken below the TWI: r${}^{2}$ = 0.329 where critical r${}^{2}$ = 0.283 at 0.05 significance.

Figure 17b, July 2015 campaign: correlation between the ground bag samples and CRDS samples: r${}^{2}$ = 0.77 where critical r${}^{2}$ = 0.171 at a 0.05 significance. This correlation is better than for September 2014 because ground samples were collected at both take-off site and co-located with the CRDS. Correlation between ground samples and samples taken below the TWI: r${}^{2}$ = 0.493 where critical r${}^{2}$ = 0.305 at 0.05 significance.

**Figure 18:**
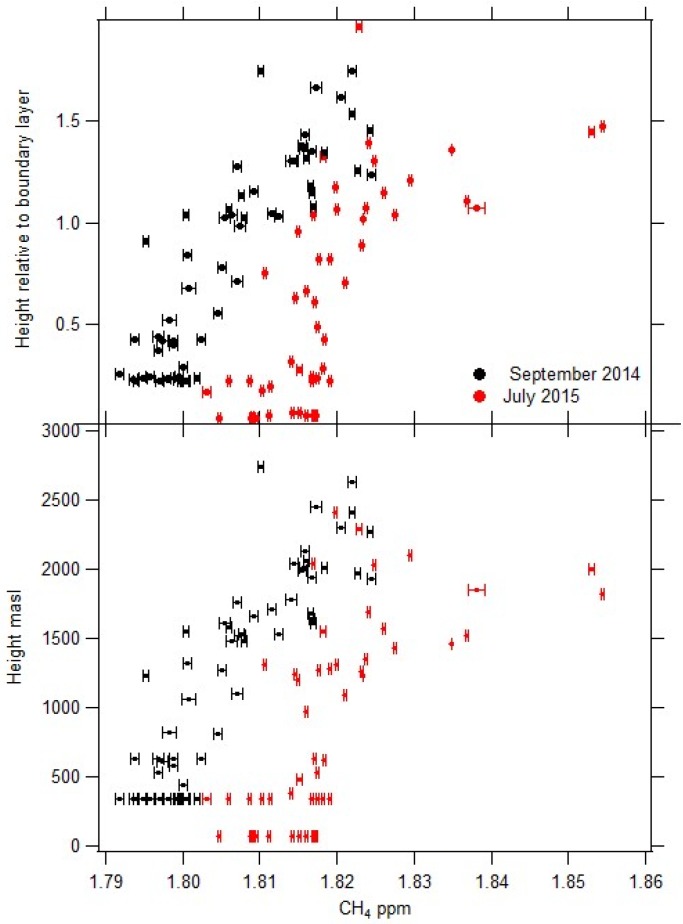
CH${}_{4}$ mole fraction (ppm) with altitude during the two campaigns (bottom). CH${}_{4}$ mole fraction normalised to the boundary layer height for the two campaigns [46].

Figure 18 shows the CH${}_{4}$ mole fraction (ppm) variation with both altitude ASL and relative to the boundary layer for both campaigns. Both the September 2014 and July 2015 campaigns show consistently higher CH${}_{4}$ mole fractions above the TWI, Figure 18, with increments up to 31 ppb. Mixed source emissions (e.g., wetlands, agriculture and biomass burning) from north of the intertropical convergence zone and Africa may be influencing the air masses. Samples from July 2015 have higher CH${}_{4}$ mole fractions and ranges compared to September 2014, which is likely to reflect the year on year growth and seasonality [46]. Different mixing ratios across the TWI may also be inferred when comparing the continuous ground level monitoring and samples with CH${}_{4}$ mole fractions above the TWI [46].

Green mountain on Ascension Island is not high enough to enable samples to be taken above the TWI without the use of an air vehicle. With the increased levels of CH${}_{4}$ shown in Figure 18, there is strong evidence a system is required in order to collect additional high altitude air samples. SUAS offers the potential for a low cost, repeatable and flexible sample system, which in the longer term could be used routinely by a non-specialist for air sample collections of this type.

## 9. Conclusions and Future Work

A multirotor has been successfully flown on Ascension Island up to 2500 m above ground level collecting air samples for analysis over the course of two campaigns in September 2014 and July 2015. The system was shown to operate reliably, amassing over one hundred flights without incidence. This paper has highlighted key aspects of the system from the vehicle specification through to flight performance data and lessons learned. Recommendations have been made for using the system to identify wind conditions at the edge of the vehicle’s flight envelope, as well as a flight profile that can alleviate issues encountered with still air.

Throughout the flights, the temperature and relative humidity were measured and transmitted to the ground station making it possible to identify the location of the trade wind inversion and the different air masses during the ascent. This real-time measurement of the atmospheric profile is a significant capability of the system and enabled the atmospheric scientists to accurately target key parts of the profile during the flights in order to collect the samples required.

Using SUAS, it has been shown that in principle greenhouse gas measurement on Ascension can access both air at ground level from a very wide swathe of the southern oceans and also sample air from above the trade wind inversion, thereby addressing emissions from a significant part of the global tropical land masses. These air samples can be used for the purposes of identifying methane mole fractions and isotopic composition.

Whilst the campaigns in September 2014 and July 2015 required a highly specialised team of engineers and scientists for successful operations, it is expected that a fully-automatic atmospheric sampling SUAS could be developed for long-term sampling requirements. This could also include the development of more capable vehicles for sampling in higher wind conditions and in more challenging environments.

One of the key requirements for the campaigns to be successful was to be able to operate the platforms Beyond Line of Sight (BLOS). The approach taken in this work was to manage the question of BLOS operations through the operational strategy, procedures and flight within restricted airspace. BLOS operations in unrestricted airspace would significantly expand the potential applications of SUAS, including air sampling; however, this integration will take time and will require significant research and development effort into platform reliability, autonomous operations, sense and avoid, as well as the legal framework. As each of these is addressed, the range of applications for which SUAS are applicable will increase, including air sampling, together with the range of airspace that is open for operations.

## Figures and Tables

**Table 1:** Key UAV specifications.

Maximum Take Off Weight (MTOW) (inc. batteries)	10 kg
Diagonal rotor-rotor distance	1.07 m
Maximum battery capacity	32,000 mAh 6-cell Lithium Polymer
Motors	T-Motor MN3515 400 KV
Propellers	T-Motor 16x5.4"
Electronic Speed Controllers (ESC)	RCTimer NFS ESC 45 A (OPTO)
Autopilot	Pixhawk by 3DRobotics
Autopilot software	ArduCopter v3.1.5
Safety pilot control link	FrSky L9R 2.4 GHz
Ground Control Station (GCS) link	Ubiquiti 5-GHz directional
Onboard computing	BeagleBone Black
Sampling pump	KNF Diaphragm pump (NMP 850 KNDC)

## Abbreviations

The following abbreviations are used in this manuscript:

AGL	Above Ground Level
ASL	Above Sea Level
BBB	Beagle Bone Black
BVLOS	Beyond Visual Line of Sight
ESC	Electronic Speed Controller
RH	Relative Humidity
RTH	Return to Home
SUAS	Small Unmanned Air System
TWI	Trade Wind Inversion
UAV	Unmanned Air Vehicle
